# Updates on Psoriasis in Special Areas

**DOI:** 10.3390/jcm13247549

**Published:** 2024-12-11

**Authors:** Alexandra-Irina Butacu, Cristian Toma, Iulia-Elena Negulet, Ionela Manole, Angela Nina Banica, Alexandra Plesea, Ioana Alexandra Badircea, Isabela Iancu, George-Sorin Tiplica

**Affiliations:** 1 2nd Department of Dermatology, Colentina Hospital, “Carol Davila” University of Medicine and Pharmacy, 050474 Bucharest, Romania; alexandra.butacu@umfcd.ro (A.-I.B.); iulia-elena.negulet@drd.umfcd.ro (I.-E.N.); ionela.manole@drd.umfcd.ro (I.M.); angela-nina.banica@rez.umfcd.ro (A.N.B.); alexandra.plesea@rez.umfcd.ro (A.P.); ioana-alexandra.badircea@rez.umfcd.ro (I.A.B.); george.tiplica@umfcd.ro (G.-S.T.); 2 3rd Department of Urology, “Prof. Dr. Theodor Burghele” Clinical Hospital, “Carol Davila” University of Medicine and Pharmacy, 050474 Bucharest, Romania; cristian.toma@umfcd.ro

**Keywords:** psoriasis, special areas, biologics, quality of life, assessment scores

## Abstract

Special areas of involvement in psoriasis include the scalp region, the palms and soles, genital areas, as well as intertriginous sites. The involvement of these topographical regions is associated with important physical and emotional implications, resulting in reduced quality of life, social isolation, and work disability. Palms and soles can be affected as part of the generalized form of psoriasis or can be exclusively affected as palmo-plantar psoriasis. Nail involvement may be encountered in 10–55% of patients with psoriasis, while scalp involvement occurs in 45–56% of individuals with psoriasis. Genital involvement may be the only manifestation of cutaneous psoriasis in 2–5% of patients. Inverse or intertriginous psoriasis represents a special variant of psoriasis as it may mimic and be difficult to differentiate from other dermatological entities that involve the intertriginous skin, such as bacterial or fungal infections, eczema, or lichen planus. Treatment of psoriasis in special areas is challenging due to the facts that special areas are more resistant to standard therapies and are more sensitive to potent local treatments. Biological therapies, proven to be more efficient than standard therapies, are not widely available in the absence of extensive skin involvement. This manuscript aims to provide an up-to-date literature review on psoriasis in special areas, benefiting the everyday clinical practice of physicians in optimizing the evaluation and treatment of their patients.

## 1. Introduction

Special areas of involvement in psoriasis include the scalp region, the palms and soles, the genital areas, as well as the intertriginous sites. The involvement of these topographical regions is associated with important physical and emotional implications, resulting in reduced quality of life, social isolation, and work disability. Psoriasis is considered more severe in these cases even if the lesions are not extensive or affect a significant proportion of the body surface area, taking into account the secondary implications as well as the frequency of resistance to treatment. These lesions are usually difficult to treat and require specific management.

Even though psoriasis in special areas is associated with a smaller area of the body surface being affected, the impact on the patient’s quality of life is significant. Therefore, in Romania, the National Protocol of Treatment in Psoriasis permits the prescription of biologic therapy in psoriasis of special areas without the assessment of lesions by severity scores such as PASI.

The pathogenesis of psoriasis in special areas presents differences from the pathogenesis of typical plaque psoriasis due to the distinct anatomical and immunological factors. In areas such as skin folds and the genital region, the specific microenvironment—characterized by higher moisture and temperature—influences local immune responses, which reduces the presence of traditional psoriatic plaques and scaling, resulting in smoother, red lesions that are more erythematous due to higher cutaneous hydration levels. In these regions, there is a predominance of interleukin (IL)-17-related pathways, which drive inflammation but differ in their cellular sources and activity levels compared to classic plaque sites. Studies indicate that inverse psoriasis has a lower expression of anti-microbial peptides and keratinocyte hyperproliferation, potentially due to a different balance of T-helper (Th) cell subsets, especially Th17 and Th1 cells, and less pronounced involvement of keratinocytes [1].

In genital psoriasis, additional stress and mechanical irritation further complicate the inflammatory response, which can exacerbate lesion sensitivity and discomfort in patients. The complex interaction between genetic predisposition, immune cell signaling (especially the IL-23/IL-17 axis), and the unique characteristics of specific areas leads to the diverse presentations of psoriasis in special regions [2].

Stress remains the most common trigger of psoriatic lesions on all body sites, even though special areas may be exposed to specific additional factors. For example, factors like friction and sweating, particularly in cases of inverse psoriasis, in association with regular skin-to-skin contact may exacerbate psoriatic lesions. Repetitive local trauma, in association with exposure to environmental factors, may represent triggering factors in cases of palmo-plantar psoriasis and nail psoriasis. Genital local trauma resulting from intercourse, as well as local moisture and frequent contact with urine and feces, may induce new lesions or may delay healing in cases of genital psoriasis [3].

The skin microbiome and its impact on the clinical findings of psoriasis lesions in different body parts is currently trending in medical studies. Psoriatic lesions may change quite substantially with regard to their microbiome, especially when it comes to special areas. Due to the local humidity, environment exposure, and skin thickness, the microbiome takes on different compositions. Scalp lesions were found to have a higher prevalence of *Staphylococcus* species, whereas *Cutibacterium acnes* is less prevalent as compared with other sites. In contrast, the moist environment provided by intertriginous folds favors the *Corynebacterium* species, which is less dominant in drier regions such as the palms and soles. These differences in the microbiome likely affect the immune response and inflammation levels and, therefore, can explain part of the variability in symptom severity and the response to treatment of various body sites [4,5].

This manuscript aims to provide an up-to-date literature review on psoriasis in special areas, benefiting the everyday clinical practice of physicians in optimizing the evaluation and treatment of their patients.

## 2. Special Areas Involved in Psoriasis

### 2.1. Palmo-Plantar Psoriasis

Palms and soles can be affected as part of the generalized form of psoriasis or can be exclusively affected as palmo-plantar psoriasis, representing 3–4% of all cases. Palmo-plantar psoriasis is more common amongst manual laborers, farmers, and housewives, taking into consideration that repetitive trauma may serve as a potential trigger [6]. Exacerbations of lesions are determined by seasonal changes and palmo-plantar psoriasis is frequently associated with psoriatic arthritis and nail psoriasis [7].

Dermoscopy may be useful in evaluating palmo-plantar psoriasis and identifying the presence of white scales and a regular arrangement of dots and globular vessels. These findings help differentiate palmo-plantar psoriasis from palmo-plantar eczema, which usually presents with yellowish scales and an irregular arrangement of atypical vessels [8]. 

Biopsy of lesions in association with histopathological examination is frequently needed as the lesions can be clinically indistinguishable from various forms of hand dermatitis [7]. Histopathological findings in palmo-plantar psoriasis often include several characteristic features that help differentiate it from other conditions. These include prominent hyperkeratosis, parakeratosis, thinning of the suprapapillary plate, and a decreased or absent granular layer. The epidermis typically shows psoriasiform hyperplasia with elongated rete ridges. Dermal features often include tortuous and dilated capillaries in the papillary dermis, with a moderate-to-intense perivascular lymphocytic infiltrate. Additionally, Munro microabscesses—clusters of neutrophils in the stratum corneum—are frequently present and serve as a hallmark of psoriasis, while spongiform pustules of Kogoj, though less common, may also be observed in palmo-plantar psoriasis cases [9].

#### 2.1.1. Clinical Presentation

**Figure 1:**
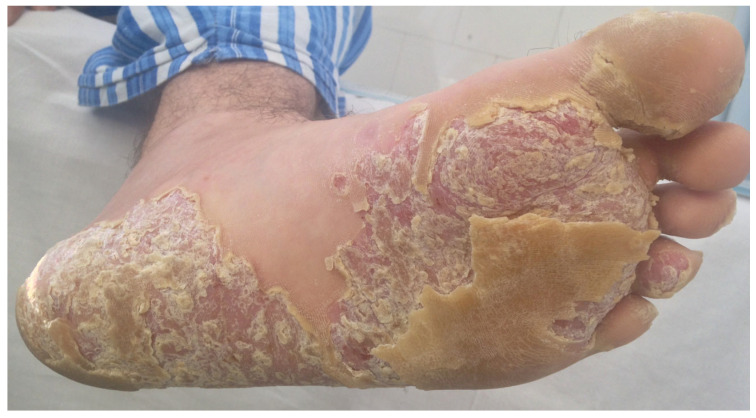
Palmo-plantar psoriasis: clinical aspects of plantar surfaces. PPPASI—21.

Palmo-plantar psoriasis usually presents hyperkeratotic and erythematous plaques with symmetrical distribution on the palmar and/or plantar regions, in association with painful fissures and nail involvement, such as pitting, subungual hyperkeratosis, and longitudinal ridging (see Figure 1—palmo-plantar psoriasis: clinical aspects of plantar surfaces). In moderate-to-severe forms, the normal elasticity of the skin underlying the finger joints is lost, causing significant functional disability [10]. The severity of skin disease activity and response to treatment is assessed through the Palmoplantar Psoriasis Area and Severity Index (PPPASI), a point-based system quantifying the area and severity of palmo-plantar psoriasis. The PPPASI measures the level of erythema, induration, and desquamation on a scale from 0 to 4 and is widely used as a measure of improvement in clinical studies [11]. Another assessment tool is represented by ESIF (erythema, scaling, induration, and fissuring), a four-point scale (from 0 = clear to 3 = severe) with a total from 0 (absence of disease) to 24 (most severe involvement) [12]. Disease clearance may also be evaluated by the Palmoplantar Psoriasis/Pustulosis Physician Global Assessment [PPPGA/PPPPGA] score, 0/1 [13].

Differential diagnoses of palmo-plantar psoriasis include palmo-plantar pustulosis, dyshidrotic eczema, contact dermatitis, pityriasis rubra pilaris, tinea pedis/manuum, and acquired palmo-plantar keratoderma [14].

#### 2.1.2. Management 

Palmo-plantar psoriasis is frequently resistant to treatment and represents a therapeutic challenge. Available data in the medical literature are limited as patients with palmo-plantar psoriasis have been perpetually excluded from clinical trials, given that the hands and feet account for less than 10% of the body surface area [15].

Topical treatment of palmo-plantar includes potent-to-superpotent topical steroids, which may be applied under occlusion, with a gradual reduction in the frequency of applications, with a duration of treatment from weeks to months. Additional topical therapies that may be used in treating palmo-plantar are represented by vitamin D analogs, calcipotriene, which may be used in association with topical steroids. One study showed that fewer than one in three patients showed improvement with topical therapy, most requiring systemic therapies. It has been hypothesized that the exaggerated thickness of the skin in the palmo-plantar regions prevents the absorption of the drugs, causing the lack of response to treatment in moderate-to-severe cases.

Phototherapy may serve as a treatment regimen of palmo-plantar, including PUVA/UVBnb/excimer laser. 

**Figure 2:**
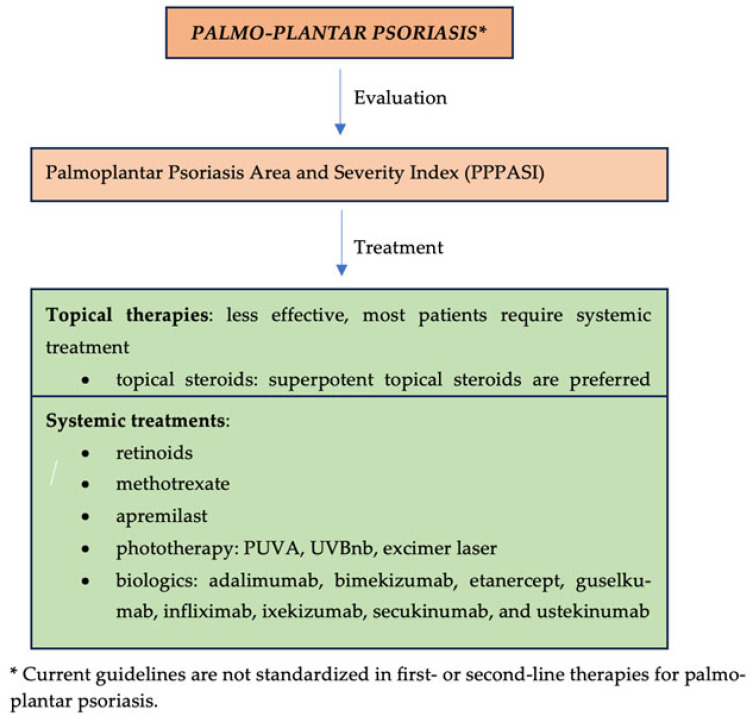
Overview of management of palmo-plantar psoriasis.

Systemic treatments are represented by retinoids (acitretin 10 mg to 50 mg per day, with a maximal effect at six months after initiation); methotrexate (7.5 mg to 20 mg per week over three to six weeks); cyclosporine, 2.5 mg/kg to 5.0 mg/kg per day for a maximum of one year; apremilast (30 mg × 2/ day); as well as biologics (secukinumab, risankizumab, guselkumab, ixekizumab, etc.) (Figure 2—overview of management of palmo-plantar psoriasis) [16,17,18,19,20,21,22]. 

Apremilast showed a reduction in the PPPGA score to 0/1 in 83.33% of cases in a cohort of 12 patients, with no significant safety issues reported in a single-center, retrospective study [21]. 

Secukinumab showed promising results in a long-term, double-blind, randomized, placebo-controlled trial specifically dedicated to patients with moderate-to-severe palmo-plantar psoriasis, assessing the efficacy and safety of secukinumab in 205 patients [22]. Ixekizumab resulted in greater and more rapid improvements than placebo and etanercept at week 12 in phase III trials and improvements were sustained with continued treatment [23]. Tildrakizumab and Risankizumab represent new treatment options that may be effective for palmo-plantar psoriasis [24,25].

A 28-week Italian retrospective study compared the efficacy of anti-IL-23s (guselkumab, risankizumab, and tildrakizumab) for 150 patients and demonstrated a comparable efficacy and safety profile for all anti-IL23, with guselkumab and risankizumab showing faster results than tildrakizumab [26].

A 2023 systematic review and network meta-analysis including 15 randomized clinical trials (RCTs) and evaluating the efficacy of adalimumab, bimekizumab, etanercept, guselkumab, infliximab, ixekizumab, secukinumab, and ustekinumab showed that all biologics except infliximab were more efficient than placebo—secukinumab showed the highest probability of determining complete resolution and ixekizumab and showed the best ranking for inducing PPPASI50 and PPPASI 75 [27].

Another network meta-analysis compared the efficacy of biologics and oral agents in PPP and palmo-plantar pustulosis, including seven RCTs, by evaluating disease clearance (PPPPGA 0/1) and improvement (PPPASI 50) in five drugs: apremilast, imsidolimab, guselkumab, spesolimab, ustekinumab. The study showed that apremilast and guselkumab were more effective than placebo in achieving PPPPGA 0/1 and PPPASI 50 [13].

### 2.2. Nail Psoriasis

Nail involvement may be encountered in 10–55% of patients with psoriasis, presenting as lesions that follow or coexist with the cutaneous psoriatic lesions or may be the single clinical manifestation of psoriasis. The etiopathogenesis involves psoriatic inflammation involving the nail unit (nail matrix and nail bed) [28]. As the nail unit is directly linked to the distal interphalangeal joint and to the extensor tendon that crosses this joint, nail psoriasis is frequently associated with psoriatic arthritis and represents an independent predictive factor for the rheumatic disease. Nail psoriasis is associated with a decreased quality of life [29].

In nail psoriasis, dermoscopy and histopathological examination reveal characteristic findings that help in diagnosis. Dermoscopically, nail psoriasis often shows pinpoint hemorrhages, irregular pitting, onycholysis with a “salmon patch” or “oil drop” discoloration, and splinter hemorrhages [30]. These features reflect inflammation in the nail matrix and nail bed, with pitting caused by defective keratinization in the proximal nail matrix and salmon patches resulting from psoriatic plaques in the nail bed. Capillary abnormalities, such as dilated blood vessels in the hyponychium and periungual regions, may also be observed under dermoscopy, particularly in more severe cases [31].

Histopathological findings in nail psoriasis include hyperkeratosis, parakeratosis, and the presence of Munro microabscesses, which are collections of neutrophils in the stratum corneum. Other notable findings are spongiform pustules and acanthosis in the nail bed, as well as thinning or absence of the granular layer in the nail matrix [32]. In cases with onycholysis, histology often reveals subungual hyperkeratosis and dilated blood vessels, which correspond to the visible hemorrhages seen dermoscopically. Together, these dermoscopic and histopathologic features support the diagnosis of nail psoriasis and help differentiate it from other nail disorders [33].

#### 2.2.1. Clinical Presentation

**Figure 3:**
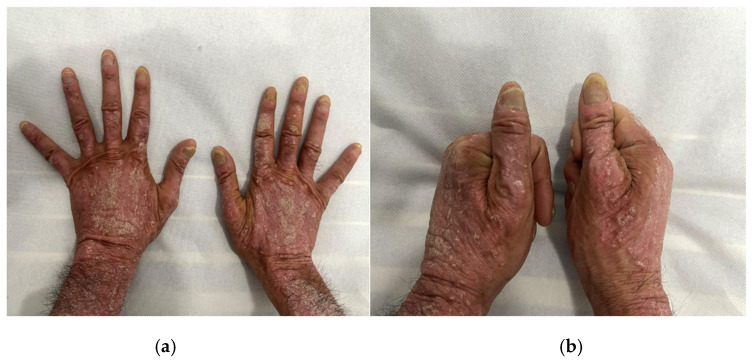
Clinical aspects of nail psoriasis: (**a**) overview of nail psoriasis; (**b**) close-up of nail psoriasis affecting the hallux. NAPSI—34.

Clinically, fingernails are more often affected than toenails and lesions differ according to the element of the nail unit that is affected. In cases of nail matrix involvement, clinical manifestations include pitting, leukonychia, red macules in the lunula, nail plate crumbling, trachyonychia, Beau lines, onychomadesis, onychodystrophy, and in the cases of nail bed involvement, lesions appear as salmon patches (oil-drop dyschromia), nail bed hyperkeratosis, splinter hemorrhages, or onycholysis (Figure 3—clinical aspects of nail psoriasis). Involvement of the lateral and proximal nail folds may also be present and may induce lesions resembling those seen in paronychia, which can affect the entire tip of the finger [29]. 

Severity scores frequently used to assess nail psoriasis include the Nail Psoriasis Severity Index (NAPSI), Nail Assessment in Psoriasis and Psoriatic Arthritis (NAPPA), and Physician’s Global Assessment of Fingernail Psoriasis (PGA-F).

NAPSI represents a useful tool in evaluating nail psoriasis, assessing any signs of disease present in the nail matrix (pitting, leukonychia, red spots in the lunula, and nail plate crumbling) or in the nail bed (oil-drop discoloration, onycholysis, hyperkeratosis, and splinter hemorrhages). Each nail is assigned a nail matrix and a nail bed score of 0–4, which are combined to yield a total score of 0–8 for each nail. All nails should be evaluated, with the total NAPSI score being the sum of the scores, up to 80 if only fingers (10 nails) are considered, or up to 160 if toes are also included (20 nails) [34]. Another useful tool is represented by the modified Nail Psoriasis Severity Index (mNAPSI) [35].

NAPPA, a modular instrument for the assessment of clinical and patient-reported outcomes in nail psoriasis, is composed of the following three elements: a questionnaire assessing the quality of life of patients (NAPPA-QoL), which is filled in by the patient; a two-part questionnaire assessing the benefits of treatment (the Patient Benefit Index, NAPPA-PBI), filled in by the patient; and the psoriasis Clinical Assessment of Severity (NAPPA-CLIN), filled in by the dermatologist.

NAPPA-QoL is a 20-item nail-specific quality-of-life questionnaire that assesses specific quality-of-life conditions in the past week. Answers are given on Likert scales from 0 to 4. Factor analysis revealed three scales named ”Signs” (nail status), “Stigma” (nail impact: stigma and emotional status), and ”Everyday life” (nail impact: everyday life). NAPPA-PBI is a 24-item questionnaire that assesses patient-defined needs before and patient-rated benefits after treatment. The answers are given on Likert scales from 0 to 4, and a global score is calculated based on the importance-weighted benefit items. NAPPA-CLIN has been developed from the NAPSI score, a nail-psoriasis-specific score, which in its original version comprises the assessment of matrix and nail bed involvement in every finger and toe by two criteria for each nail. The NAPPA-CLIN is a simplified version of the NAPSI, which only assesses the least and the worst involved nails of both hands or both feet, respectively [36]. 

PGA-F represents a clinician-rated severity instrument that can be applied in clinical practice and research activities. PGA-F synthesizes severity ratings across different aspects of psoriatic nail disease and yields an overall severity score that classifies individuals into one of five clinically meaningful and distinct categories: clear, minimal, mild, moderate, and severe [37].

The Severity of Nail Psoriasis Score (SNAPS) assesses the presence of four features of fingernail psoriasis, pitting, onycholysis, hyperkeratosis, and severe nail deformity, with a score from 0 to 40 [38].

Differential diagnoses of nail psoriasis include onychomycosis, lichen planus, alopecia areata, pityriasis rubra pilaris, and nail trauma [29].

#### 2.2.2. Management

General measures of treatment in nail psoriasis are represented by the avoidance of local trauma, wearing protective gloves, gentle washing of the nails and the periungual folds, keeping the nails trimmed for facilitating the application of local treatment, as well as properly hydrating the periungual folds. 

In cases of mild nail disease, first-line therapy is represented by topical calcipotriol in association with high-potency topical corticosteroid or high-potency topical corticosteroid or topical calcipotriol; the second-line therapy includes topical tacrolimus and topical tazarotene. In cases refractory to topical therapy, options reserved for moderate-to-severe disease should be considered [39]. 

In moderate-to-severe nail psoriasis, first-line therapy typically includes biological agents targeting specific inflammatory pathways. Tumor necrosis factor (TNF)-alpha inhibitors, such as adalimumab, etanercept, infliximab, certolizumab pegol, and golimumab, have demonstrated moderate-to-high efficacy in treating psoriatic nail disease [40]. Recent studies on the treatment of nail psoriasis highlight the significant progress in biologic therapies, with newer agents like IL-17 and IL-23 inhibitors showing promising results in terms of efficacy and speed of improvement. Notably, the IL-17 inhibitor bimekizumab, which targets both IL-17A and IL-17F, demonstrated rapid and substantial improvements [41]. A multicenter study from Italy involving over 800 patients showed that by 36 weeks, 88.5% of patients treated with bimekizumab achieved complete clearance of nail psoriasis, with considerable reductions in psoriasis severity and improved quality-of-life scores. This rapid response underscores the potential benefit of dual IL-17 inhibition over traditional therapies that target single cytokines, particularly in achieving faster nail improvement and mitigating progression to psoriatic arthritis [42]. Brodalumab, an IL-17 receptor A antagonist, has shown efficacy in treating nail psoriasis, with recent phase III trials indicating substantial improvements in nail symptoms among patients with moderate-to-severe plaque psoriasis. These trials demonstrated that brodalumab significantly reduces inflammation and leads to clinical improvement in challenging areas like the nails and scalp [43]. Comparative studies have shown that IL-17 inhibitors generally offer superior efficacy to IL-12/23 inhibitors, such as ustekinumab, for nail psoriasis. IL-23 inhibitors like guselkumab and risankizumab have also performed well but tend to have a slower onset of action compared to IL-17 agents. In another head-to-head comparison, guselkumab and adalimumab were effective for nail psoriasis but with slightly slower clearance rates than bimekizumab. This places IL-17 inhibitors, especially those targeting dual pathways, as preferred options for patients with moderate-to-severe nail involvement where rapid symptom relief is a priority [44].

**Figure 4:**
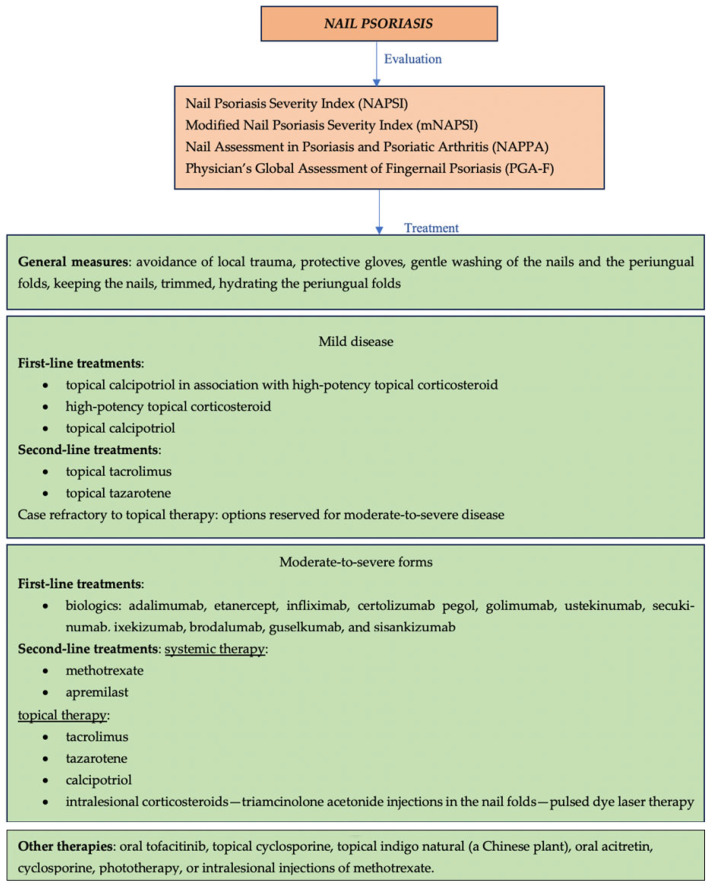
Overview of management of nail psoriasis.

Second-line therapy includes methotrexate, topical therapy (tacrolimus, tazarotene, and calcipotriol), intralesional corticosteroids—triamcinolone acetonide injections in the nail folds—and pulsed dye laser therapy. Other therapies may be used, including oral tofacitinib, topical cyclosporine, topical indigo natural (a Chinese plant), oral acitretin, and cyclosporine, phototherapy, or intralesional injections of methotrexate (Figure 4—overview of management of nail psoriasis) [39]. Apremilast, an oral phosphodiesterase-4 (PDE4) inhibitor, has shown efficacy in the treatment of nail psoriasis and may be used as second-line therapy. Studies within the last two years indicate that apremilast can achieve significant reductions in the Nail Psoriasis Severity Index (NAPSI) score, a measure of severity, in patients with nail involvement. In trials such as ESTEEM 1 and 2, up to 55.4% of patients achieved a NAPSI-50 response (a 50% improvement in nail severity) at week 32 [45].

### 2.3. Scalp Psoriasis

Scalp involvement occurs in 45–56% of individuals with psoriasis. The scalp is typically one of the first affected areas of the body, with the frequency of lesion formation increasing with disease duration. The quality of life of patients may be decreased by pain, itching, bleeding, feelings of embarrassment, and restricting clothing choices [15].

Trichoscopy is a diagnostic method widely used for the differential diagnosis of hair loss. Recently, several research articles addressed the possible application of trichoscopy in inflammatory scalp diseases. Trichoscopy in scalp psoriasis was evaluated in a total of 12 studies—8 original studies and 4 case reports—including a total of 218 patients and additionally 48 lesions. The most common vessel type observed in the interfollicular region were twisted red loops, glomerular vessels, and red dots and globules.

White, perifollicular scaling was observed in 109/218 (50%) patients but in 48/48 (100%) lesions reported by Lallas et al., yellow scaling was rarely reported. The distribution of the scaling was described as diffuse or patchy. The most common other dermoscopic features included punctate hemorrhage, blotchy erythema, and structureless red areas [46].

#### 2.3.1. Clinical Presentation

**Figure 5:**
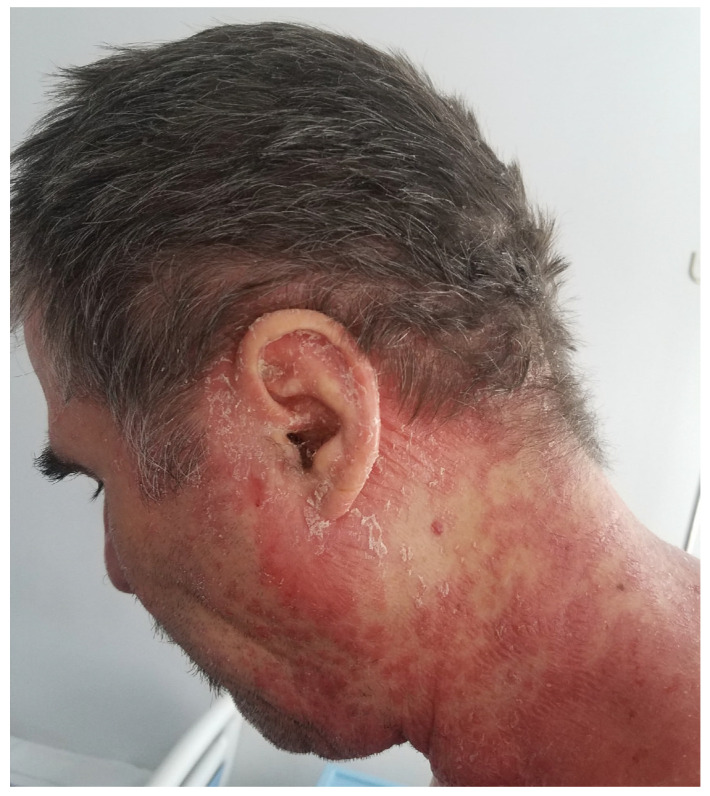
Scalp psoriasis: scalp involvement in a patient with psoriasis vulgaris. PSSI—29.

Scalp lesions are characterized by thickened, well-demarcated, squamous, erythematous plaques, frequently associated with itching. The lesions are typically located on the retro-auricular and cervical regions, even though they may appear anywhere on the scalp. The extent of the disease varies from fine scaling to thick erythematous crusted plaques on the entire scalp, typically crossing the hair line and affecting a small area of the adjacent facial skin. In severe cases, alopecia due to psoriatic plaques has been reported (Figure 5—scalp psoriasis: scalp involvement in a patient with psoriasis vulgaris) [47]. 

The severity of scalp psoriasis is assessed through the Psoriasis Scalp Severity Index (PSSI), which evaluates erythema, infiltration, and desquamation on a scale from 0 to 4 and calculates the percentage of affected scalp in six categories. Videodermoscopy associated with PSSI (VSCAPSI) may also be a useful tool for the evaluation of scalp psoriasis, especially in mild and moderate forms. VSCAPSI provides better visualization of the scalp area affected by psoriasis, the presence and morphology of vascular patterns, as well as erythema and desquamation [48,49].

Differential diagnoses of scalp psoriasis include seborrheic dermatitis, tinea capitis, and lichen planopilaris [50]. Seborrheic dermatitis is defined by patches that vary from pink-yellow to red-brown, surmounted by flaky greasy scales. It predilects the areas rich in sebaceous glands such as the scalp, face, ears, and presternal region. In seborrheic dermatitis, there is erythema in addition to dandruff. Toward the forehead, erythema and scaling are usually sharply demarcated from uninvolved skin, with the border either at the hairline or slightly transgressing beyond it. Distinction of seborrheic dermatitis from psoriasis could be difficult and there may be an overlap in some patients, such as those with sebopsoriasis. However, the plaques of psoriasis tend to be thicker, with silvery-white scales that are more discrete and not associated with seborrhoea. When a differential diagnosis is not easily made on a clinical basis, video capillaroscopy could be a useful non-invasive approach for differentiating between psoriasis and seborrheic dermatitis, especially when the scalp is the only affected site. Scalp psoriasis exhibits homogeneously tortuous and dilated capillaries (bushy pattern) with a larger diameter than seborrheic dermatitis [14]. A retrospective observational study evaluated 15 cases of psoriasis and 20 cases of seborrheic dermatitis of the scalp and summarized that histopathological features indicating psoriasis include mounds of parakeratosis with neutrophils, spongiform micropustules of Kogoj, clubbed and even lengths of rete ridges, and increased numbers of mitotic figures (≥6/HPF). Features favoring seborrheic dermatitis are follicular plugging, shoulder parakeratosis, and prominent lymphocytic exocytosis. Immunohistochemistry including Ki-67, keratin 10, caspase-5, and GLUT-1 was not helpful in differentiating psoriasis from seborrheic dermatitis of the scalp [51]. Tinea capitis is a disease caused by a fungal infection of the skin, hair shafts, and follicles of the scalp. It is usually more circumscribed and less diffuse than seborrheic dermatitis, or psoriasis associated with hair loss or broken-off hairs. Tinea capitis occurs almost exclusively in children and is very rare in adults [14]. Lichen planopilaris is a chronic inflammatory disorder characterized by follicular and perifollicular scaly and pruritic papules on the scalp. These lesions usually progress over time to atrophic cicatricial alopecia. This disorder is more common in women than men and may be associated with ungual and erosive mucosal involvement. A variant of lichen planopilaris is frontal fibrosing alopecia. It is characterized by marginal progressive hair loss on the scalp, eyebrows, and axillae. Skin histological examination of the scalp lesions is diagnostic. Common findings include lichenoid lymphocyte infiltration in the follicular dermo-epidermal junction, wedge-shaped hypergranulosis, colloid bodies, loss of sebaceous glands, and the destruction of hair follicle root sheaths and follicular plugging. Lichen planopilaris may coexist with psoriasis, albeit rarely [14].

#### 2.3.2. Management 

First-line therapies used in the management of scalp psoriasis include topical steroids, vitamin D analogs in association with topical steroids, keratolytics, as well as systemic agents. Topical corticosteroids are preferred in cases of scalp involvement without other sites affected. In scalp psoriasis, topical steroids should be used as foam, gel, solution, shampoo, or spray [52]. Topical steroids in association with vitamin D analogs have shown long-term results. Keratolytic agents may be used for scalp disease with minimal body involvement and may be used as monotherapy or in combination with a topical steroid; however, these agents are challenging to use on the scalp because hair makes the application burdensome, and many patients find them cosmetically unacceptable. These issues can lead to non-adherence and dissatisfaction with treatment options [15].

Systemic therapies include methotrexate, cyclosporine, acitretin, biologics, and phosphodiesterase-4 inhibitors. Systemic agents represent the first line of treatment for patients with scalp psoriasis and accompanying moderate-to-severe psoriasis of the trunk and extremities [53].

A large proportion of patients with severe scalp psoriasis presents with minimal chronic plaque psoriasis on the body and, hence, may not receive systemic therapy indicated for moderate-to-severe chronic plaque psoriasis. The adjunctive use of keratolytic and tar-based shampoos, along with topical therapy (steroids and vitamin D analogs), may suffice initially but long-term compliance can be poor. Localized ultraviolet B therapy using fiber-optic hair brushes is successful for patients but these devices are expensive and patients need high allowances on their durable medical equipment insurance to obtain them. Use of methotrexate and biologics is much needed in these patients. However, approval through third-party payers can be difficult as current on-label indications require significant body-surface-area coverage or a high enough PASI score to qualify for these systemic agents [15].

It is difficult to determine the most effective biologic or small molecule in the treatment of scalp psoriasis given the varying differences in outcome measures and lack of head-to-head studies. Guselkumab, infliximab, ixekizumab, and brodalumab appear, on average, to have the highest efficacy in the clearance of scalp psoriasis across studies. However, only guselkumab, secukinumab, and apremilast have scalp psoriasis efficacy evidence from the Food and Drug Administration (FDA) [54]. Among biologics, ixekizumab has demonstrated higher long-term efficacy rates [55].

An international, prospective, non-interventional, study(PSoHO) compared the effectiveness of anti-interleukin (IL)-17A biologics (ixekizumab and secukinumab) to other approved biologics and the pairwise comparative effectiveness of ixekizumab relative to five other individual biologics for patients with moderate-to-severe psoriasis. Of the 1978 patients included, 83.4% had at least one special area involved at baseline, with the scalp (66.7%) the most frequently affected part, followed by nails (37.9%), face/neck (36.9%), genitalia (25.6%), and palms and/or soles (22.2%). Patients with scalp, nail, or genital but not palmo-plantar or face/neck psoriasis had significantly higher odds of achieving clearance at week 12 in the anti-IL-17A cohort compared to the other biologics cohort. Patients with scalp psoriasis had a 10–20% higher response rate and significantly greater odds (1.8–2.3) of achieving clearance at week 12 with ixekizumab compared to included biologics [56].

**Figure 6:**
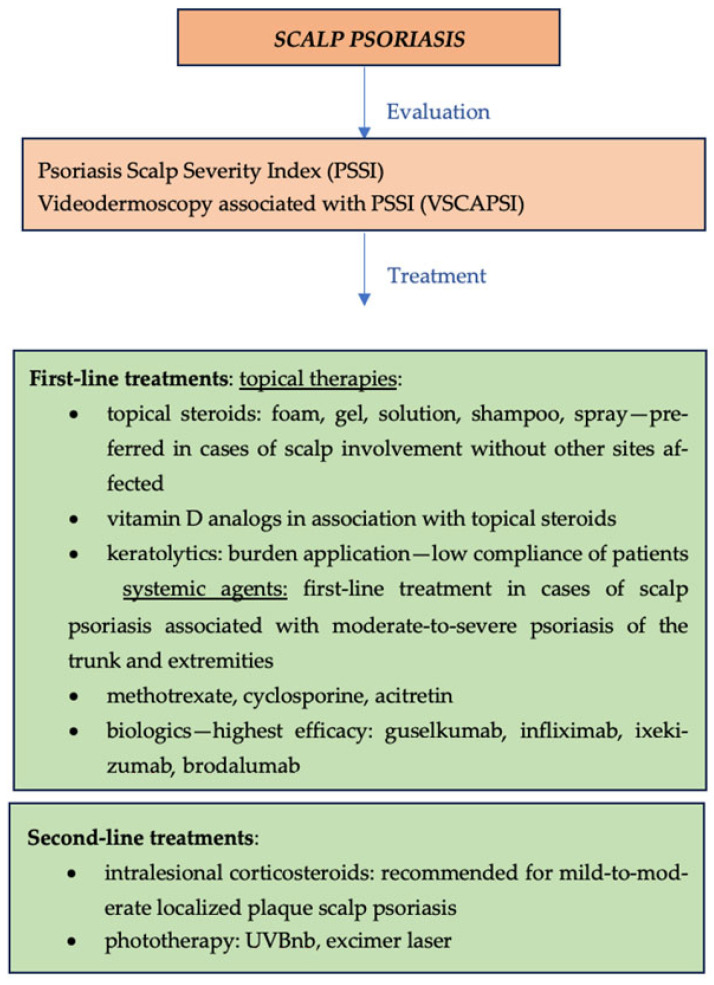
Overview of management of scalp psoriasis.

Second-line therapies include intralesional corticosteroids and phototherapy. Intralesional corticosteroids are recommended for mild-to-moderate localized disease and are especially effective for localized psoriasis plaques of the scalp. Phototherapy is preferred in cases of scalp psoriasis of any severity, with minimal body involvement. UVB narrow band and 308-nanometer excimer laser represent effective options for the management of scalp psoriasis. Faster improvements in both scalp severity and quality of life may be seen with cases treated by UVA1 phototherapy, even though there is a paucity of data on scalp psoriasis and its use is limited by the lack of availability of handheld devices (Figure 6—overview of management of scalp psoriasis) [53].

### 2.4. Genital Psoriasis

Genital involvement may be the only manifestation of cutaneous psoriasis in 2–5% of patients. Underdiagnosis, from both the healthcare providers and patients, determines an inadequate evaluation of genital psoriasis and an inefficient management of these cases. Genital psoriasis is associated with a significant negative impact on the quality of life of patients, their psychosexual well-being, and their sexual health [57].

The most frequently involved areas in genital psoriasis are represented by the penis shaft and the scrotum, while the glans, the labia majora, and the perineum in females are less frequently involved. In female patients, genital psoriasis is associated with a higher rate of symptomatology, while male patients experience decreased sexual function without erectile dysfunction [58].

Dermoscopic examination of genital psoriatic lesions reveals a distinct vascular pattern that is not commonly observed in other types of inflammatory genital diseases. It consists of either dilated and tortuous capillaries that appear in a homogenous, “bushy” structure or as dotted vessels. The appearance of these vessels may vary depending on the magnification used but they remain regularly distributed over a light red or pink background. This vascular pattern can aid in differentiating genital psoriasis from other inflammatory genital dermatoses, where the vessels are not distributed as regularly [59].

The histopathological correspondence of these dermoscopic findings is the presence of dilated, elongated, and tortuous capillary loops within the papillary dermis. Unlike psoriatic lesions in other areas of the body, this type of psoriasis generally lacks in scaling, a common finding associated with psoriasis [60].

Management of genital psoriasis is challenging due to the sensitive and fragile genital skin, which is prone to a higher risk of local side effects. The genital skin is thinner and frequently occluded, which increases absorption of topical treatments, leading to a higher potency and side effects such as atrophy, telangiectasia, and striae [58].

#### 2.4.1. Clinical Presentation

**Figure 7:**
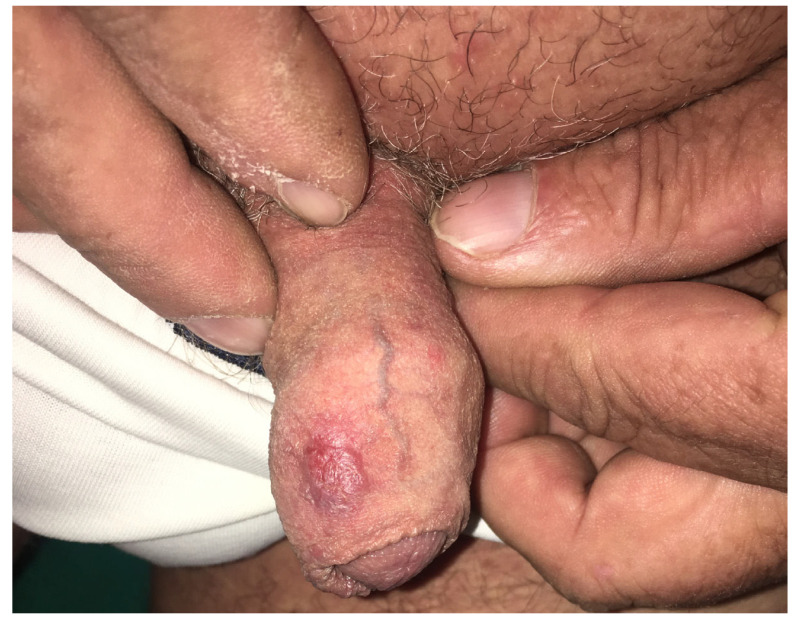
Psoriasis plaque of the genital region in a male patient. sPGA-G—3 (moderate).

Genital psoriasis is clinically characterized by well-demarcated, erythematous, thin plaques with variable degrees of scaling. The specific features of the genital region, including warmth and moisture, are responsible for the local maceration and reduction in scaling. The most frequently reported symptom in genital psoriasis is pruritus, being an important debilitating symptom in many cases. Other reported clinical manifestations are represented by burning sensations and dyspareunia (Figure 7—psoriasis plaque of the genital region in a male patient) [57].

Assessment of genital psoriasis is performed through the Static Physician’s Global Assessment of Genitalia since the PASI score has limitations with capturing the involvement of the genital areas. Clinical findings such as erythema, induration, and scaling are evaluated and scored on a 0–5 scale and the final score is associated with a category of severity: clear, minimal, mild, moderate, severe, or very severe [61]. The Genital Psoriasis Symptoms Scale (GPSS) measures the intensity of symptoms in genital psoriasis. The GPSS evaluates the clinical findings of itching, pain, discomfort, stinging, burning, redness, scaling, and cracking, and each item receives a score from 0 to 10, providing results that range from 0 to 80. The main advantage of GPSS is represented by the fact that patients feel more comfortable to communicate their symptoms through this type of questionnaire than through a face-to-face conversation [62].

Differential diagnoses of genital psoriasis in female patients include atopic dermatitis, contact dermatitis, lichen sclerosus, lichen ruber planus, and vulvar (pre)malignant lesions, and in male patients, irritative balanitis, zoon’s balanitis, erythroplasia of Queyrat, and extramammary Paget [63].

#### 2.4.2. Management

General measures used in the management of genital psoriasis include good hygiene and reduction in friction. Gentle, non-soap cleansers are recommended to keep the genitals clean without irritating the area. Furthermore, patients should be advised to wear loose-fitting, unrestrictive clothing to avoid koebnerization and further irritation [64].

Topical therapies include topical steroids, especially low-potency topical steroids, administrated for a short period of time (2–4 weeks) due to possible adverse reactions; moderate-to-strong steroids agents in severe cases; and vitamin D analogs prescribed in combination with topical steroids [63]. Other topical treatments are represented by topical calcineurin inhibitors, which are efficient for the thinner skin of intertriginous areas; antifungal agents, which may be needed in order to reduce the microbial colonization; and crisaborole, a non-steroidal topical agent approved for the treatment of mild-to-moderate atopic dermatitis (a double-blind, randomized, vehicle-controlled trial demonstrated efficiency and safety of crisaborole 2% ointment in the treatment of intertriginous, genital, and facial psoriasis) [64]. Recently, roflumilast 0,3% cream has been approved by the FDA for the treatment of sensitive area psoriasis such as the intertriginous areas. Daily use of one application on the lesions showed improvements within 6 weeks and less adverse effects in comparison with low-to-mid-potency steroids and vitamin D analogs [65].

**Figure 8:**
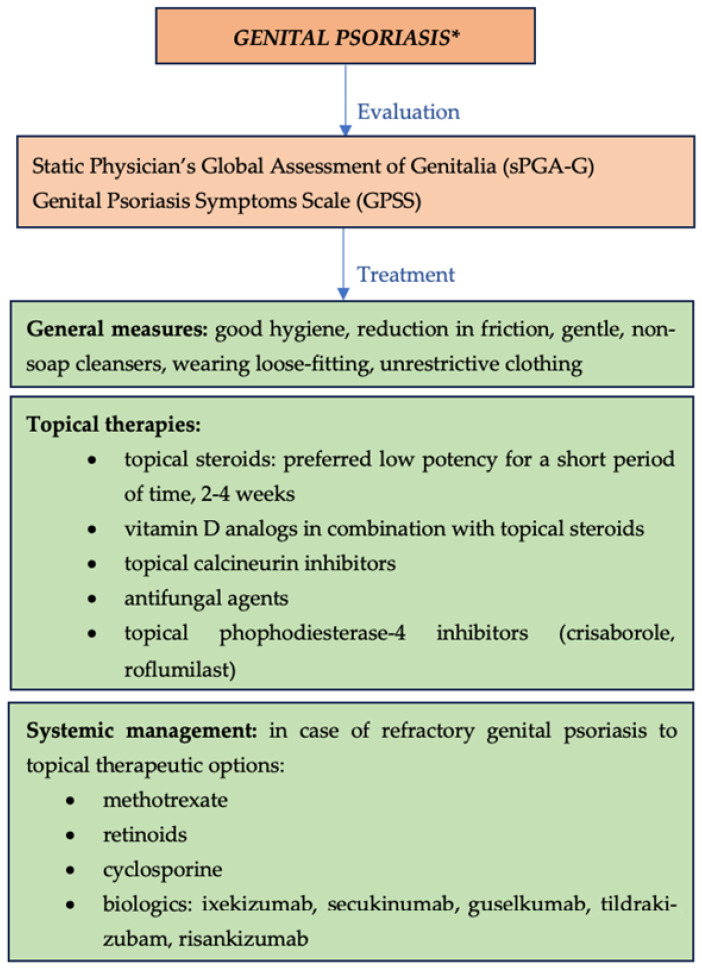
Overview of management of genital psoriasis. * Current guidelines are not standardized in first- or second-line therapies for genital psoriasis.

Systemic management is recommended in cases of refractory genital psoriasis to topical therapeutic options. The main systemic agents used in the management of genital psoriasis include methotrexate, retinoids, cyclosporine, and biological agents. Ixekizumab was proven to be efficient in treating genital psoriasis in a randomized, double-blinded, placebo, controlled phase III trial [66]. An open-label randomized controlled study compared the efficacy and safety of ixekizumab versus secukinumab in genital psoriasis, resulting in a clinical response appearing in the first two weeks of treatment, which was similar between the two drugs [64]. In another randomized clinical trial, ixekizumab and secukinumab were shown to reduce genital psoriasis symptoms and the impact on sexual intercourse [67]. Guselkumab, tildrakizumab, and risankizumab are valid options for psoriasis management, including difficult-to-treat regions, such as the genital area (Figure 8—overview of management of genital psoriasis) [24]. There was no significant difference in the clinical response to therapy between IL-23 and IL-17 inhibitors in a single-center retrospective comparative study from 2023 [68].

### 2.5. Inverse Psoriasis

Inverse psoriasis (flexural or intertriginous psoriasis) is a clinical variant of psoriasis that involves the intertriginous areas [69]. This subtype has been termed “inverse” because it affects the flexural surfaces, whereas the typical plaque psoriasis findings are located on the extensor areas. The prevalence of inverse psoriasis ranges from 3 to 36% [70]. It is common in young infants as it affects the diaper area (“napkin psoriasis”) [71]. Fungal (especially candidal) and bacterial colonization of these sites may represent a potential trigger factor [70]. It has also been noted that the abrupt onset of inverse psoriatic lesions in adults can indicate underlying HIV infection [69].

Dermoscopy could enhance the diagnosis of inverse psoriasis. The typical vascular structures can be easily identified because of the scale absence: red dots (seen as “bushy capillaries” in higher magnification) being homogeneously distributed within the erythematous background [72]. 

The diagnosis of inverse psoriasis is sometimes difficult to establish, especially when these areas are exclusively involved. A skin biopsy is then required to differentiate inverse psoriasis from other clinically similar diseases. The histopathologic findings of inverse psoriasis consist of epidermal hyperplasia, with acanthosis and parakeratosis. The rete ridges appear elongated and the granulosis layer shows a reduction in thickness [69]. Spongiosis is more common and epidermal hyperplasia is less marked in comparison to classical plaque psoriasis. Occasionally, spongiform pustules of Kogoj and Munro microabcesses can be seen [70].

#### 2.5.1. Clinical Presentation

**Figure 9:**
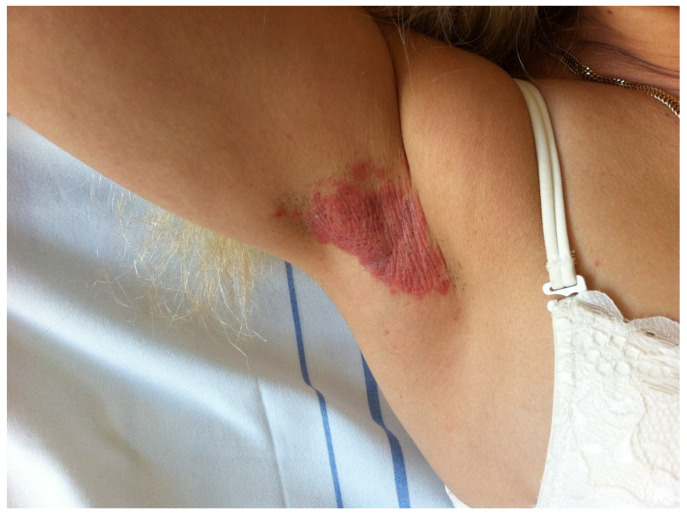
Inverse psoriasis—smooth, shiny psoriasis plaque of the axillary region. PASI—0.6.

The most common sites involved are the inguinal folds (most commonly), inframammary folds, the perianal area, retroauricular area, axilla, and umbilicus [69]. Antecubital and popliteal fossae can also be affected [70]. The lesions consist of sharply demarcated erythematous plaques, with a shiny appearance, smooth surface, and lack the typical silvery scale (Figure 9—smooth, erythematous, shiny psoriasis plaque of the axillary region). A central fissure is often seen. Flexural psoriatic lesions can present with maceration [73]. Due to local friction, the Koebner phenomenon is often described [70]. Regular plaque psoriasis lesions found in other body areas can accompany flexural findings. Superficial erosions are often associated, leading to consequent pruritus, a burning sensation, and irritation [15].

The assessment of inverse psoriasis may be conducted through PASI. Another useful tool used for evaluating the burden associated with inverse psoriasis is represented by the Inverse Psoriasis Burden of Disease (IPBOD) questionnaire, which evaluates aspects related to the quality of life of patients with inverse psoriasis [74].

Differential diagnoses of inverse psoriasis include mechanical intertrigo, bacterial or fungal infections, irritant or allergic contact dermatitis, lichen planus, atopic dermatitis, Darier disease, Hailey–Hailey disease, and extramammary Paget’s disease [69].

#### 2.5.2. Management

Inverse psoriasis is often difficult to treat as these special regions are prone to adverse reactions and are impossible to use high-potency local therapies in [75].

First-line options include low-tomid-potency topical corticosteroids. Fluticasone propionate 0.005%, a mid-strength topical steroid used twice daily for 2 weeks, has shown more than 50% improvement in facial and intertriginous psoriatic lesions. The results were maintained for 8 more weeks with a once-daily application for 2 consecutive days every week. Due to the possibility of percutaneous absorption and the occlusive nature of the flexural areas, leading to increased penetration, short-term therapy is required (maximum of 4 weeks) [76]. Topical calcineurin inhibitors are an additional therapy option for inverse psoriasis. Tacrolimus and pimecrolimus can be used as long-term therapy. The use of topical calcineurin inhibitors for the treatment of inverse psoriasis has been supported by many studies, one of which demonstrated that applying tacrolimus ointment 0.1% twice daily achieved clearance or significant improvement after as early as 8 days of treatment [77]. Topical vitamin D analogs (calcipotriol and calcitriol) can be successfully used as a long-term treatment. Studies have shown that topical vitamin D analogs are less effective compared to topical corticosteroids or topical calcineurin inhibitors [78].

Second-line options consist of topical treatments such as emollients, tar-based products, and topical antibacterial and antifungal agents. Coal tar 2% and topical salicylate applied twice daily showed significant improvement at week 8 for one patient, with some post-inflammatory hyperpigmentation [79]. Topical antifungal and antibacterial preparations have been used to treat flares of inverse psoriasis. Severe cases of inverse psoriasis or cases that are resistant to topical treatment are candidates for systemic conventional treatment: methotrexate, cyclosporine, and mycophenolate mofetil. Methotrexate should be limited to patients with debilitating quality-of-life impairment [78]. Tofacitinib, a JAK 1/3 inhibitor, has been successfully used in a patient with inverse psoriasis, alopecia areata, and vitiligo. The patient also underwent NB-UVB sessions, supporting the efficacy of JAK inhibitors used in conjunction with phototherapy [80]. Biologic therapies have also been described as useful in treating inverse psoriasis. Ixekizumab, a monoclonal antibody targeting IL-17A, is currently the only agent that demonstrates improvement in inverse psoriasis lesions. A randomized, phase III clinical trial confirmed the long-term efficacy and safety of ixekizumab for up to 52 weeks. A total of 73% of patients were reported to achieve clearing or almost clearing of the lesions by the end of week 12 [81]. Two other biologic therapies were reported as treatments for flexural psoriasis. A case report pictured successful treatment of inverse psoriasis with Adalimumab (anti-TNF-alpha agent) [82]. In another case report, a patient was initiated with Ustekinumab (IL 12/23 inhibitor), with significant improvement in pruritus, erythema, and quality of life [83].

**Figure 10:**
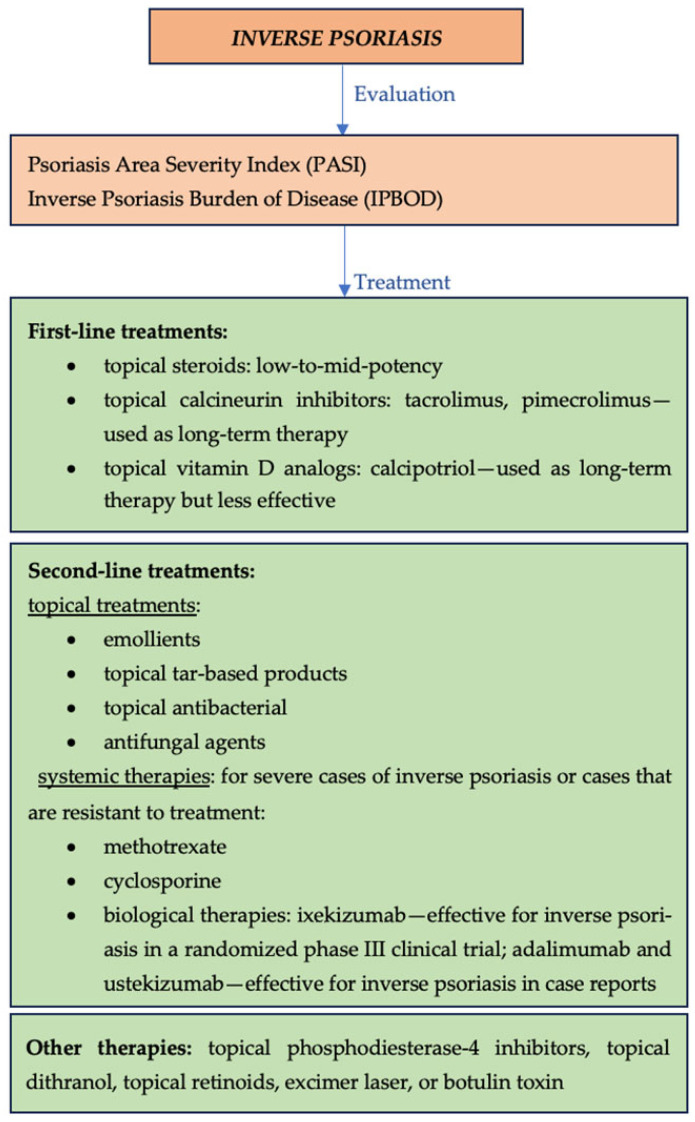
Overview of management of inverse psoriasis.

Other treatment options for inverse psoriasis include topical phosphodiesterase-4 inhibitors (crisaborole), oral PDE-4 inhibitor (apremilast), monochromatic excimer light, and botulinum toxin type-A (BoNTA) injection [76] (Figure 10).

## 3. Conclusions

Psoriasis of special areas is associated with a significant impact on the patient’s quality of life. Genital psoriasis has an important impact on daily activities but also on personal relationships, work, and emotions. Nail or hand/sole psoriasis can cause increased financial burdens due to reduced workplace productivity. Physicians need to pay proper attention to these locations in order to provide adequate care. These special areas of psoriasis require appropriate treatment as they are more resistant to standard therapies and more sensitive to potent local treatments. Biological therapies, proven to be more efficient than standard therapies, are not widely available in the absence of extensive skin involvement.
